# Eosinophilic granuloma of the cervical spine manifesting as torticollis in a child

**DOI:** 10.11604/pamj.2014.19.36.3970

**Published:** 2014-09-16

**Authors:** Ali Akhaddar, Mohamed Boucetta

**Affiliations:** 1 Department of Neurosurgery, Avicenne Military Hospital, Marrakech, Morocco; 2 University of Mohammed V Souissi, Rabat, Morocco

**Keywords:** Eosinophilic granuloma, ervical spine, torticollis

## Image in medicine

A 10-year-old boy presented with torticollis and neck pain for 2 months without fever. His physical examination showed torticollis and limitation of flexion/extension and cervical rotation without neurological deficit. Family and past histories were unremarkable. Cervical spine radiographs showed a cervical scoliosis with loss of the normal cervical lordosis and partial collapse of C3 vertebral body (A and B). Cervical computed tomography-scan and magnetic resonance imaging demonstrated a destruction of C3 vertebral corpus (vertebra plana) without discitis or spinal cord compression (C and D). A malignant bony tumor was suspected. Routine blood tests showed a mildly elevated erythrocyte sedimentation rate. Other skeletal X-rays revealed a well-defined solitary metaphyseal lytic lesion in the medial femoral condyle (E). An anterior cervical corporectomy was performed and the spine was stabilised with a tricortical iliac crest graft with plate/screws fixation (F). Histological features were consistent with eosinophilic granuloma. The patient was discharged home pain free with a good outcome. Eosinophilic granuloma (EG) is a benign and solitary bony lesion of unknown etiology. EG, Letterer-Siwe, and Hand-Schuller-Christian disease represent a spectrum of the same disease entity now known as Langerhans cell granulomatosis or histiocytosis X. EG predominantly affects the skull, the ribs, the pelvis, the mandible, and the metaphyses of other long bones. Although rare, EG should always be included in the differential diagnosis for osteolytic lesions of the spine in children.

**Figure 1:**
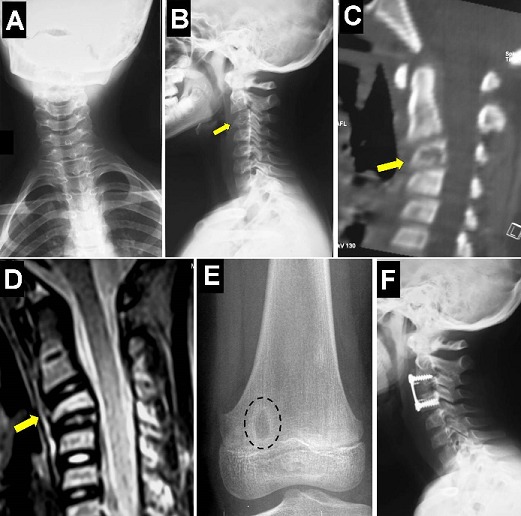
Cervical spine X-rays showing a cervical scoliosis with loss of the normal cervical lordosis and partial collapse of C3 vertebral body (A and B). Sagittal reformatted CT-scan and sagittal T2-weighted MR imaging demonstrating the C3 vertebral corpus destruction (vertebra plana) (arrows) without discitis or spinal cord compression (C and D). Simple left knee X-ray (anteroposterior view) revealing a well-defined solitary metaphyseal lytic lesion in the medial femoral condyle (E). Cervical spine X-ray (lateral view) showing the normal cervical lordosis and the anterior fixation plate (F)

